# Interplay between TAp73 Protein and Selected Activator Protein-1 (AP-1) Family Members Promotes AP-1 Target Gene Activation and Cellular Growth*

**DOI:** 10.1074/jbc.M115.636548

**Published:** 2015-05-27

**Authors:** Deepa Subramanian, Wilawan Bunjobpol, Kanaga Sabapathy

**Affiliations:** From the ‡ Division of Cellular and Molecular Research, Humphrey Oei Institute of Cancer Research, National Cancer Centre, 11 Hospital Drive, Singapore 169610, Singapore,; § Cancer and Stem Cell Biology Program, Duke-NUS Graduate Medical School, 8 College Road, Singapore 169857, Singapore, and; ¶ Department of Biochemistry, National University of Singapore, 8 Medical Drive, Singapore 117597, Singapore

**Keywords:** AP1 transcription factor (AP-1), c-Fos, c-Jun transcription factor, cell growth, p73, cellular survival, Fra1, gene transcript

## Abstract

Unlike p53, which is mutated at a high rate in human cancers, its homologue p73 is not mutated but is often overexpressed, suggesting a possible context-dependent role in growth promotion. Previously, we have shown that co-expression of TAp73 with the proto-oncogene c-Jun can augment cellular growth and potentiate transactivation of activator protein (AP)-1 target genes such as cyclin D1. Here, we provide further mechanistic insights into the cooperative activity between these two transcription factors. Our data show that TAp73-mediated AP-1 target gene transactivation relies on c-Jun dimerization and requires the canonical AP-1 sites on target gene promoters. Interestingly, only selected members of the Fos family of proteins such as c-Fos and Fra1 were found to cooperate with TAp73 in a c-Jun-dependent manner to transactivate AP-1 target promoters. Inducible expression of TAp73 led to the recruitment of these Fos family members to the AP-1 target promoters on which TAp73 was found to be bound near the AP-1 site. Consistent with the binding of TAp73 and AP-1 members on the target promoters in a c-Jun-dependent manner, TAp73 was observed to physically interact with c-Jun specifically at the chromatin via its carboxyl-terminal region. Furthermore, co-expression of c-Fos or Fra1 was able to cooperate with TAp73 in potentiating cellular growth, similarly to c-Jun. These data together suggest that TAp73 plays a vital role in activation of AP-1 target genes via direct binding to c-Jun at the target promoters, leading to enhanced loading of other AP-1 family members, thereby leading to cellular growth.

## Teaser

**Background:** TAp73, which is overexpressed in cancers, activates AP-1 target genes.

**Results:** TAp73 binds to c-Jun on the chromatin around TRE sites on AP-1 target promoters, leading to recruitment of other AP-1 family members.

**Conclusion:** Interaction of TAp73 with selected AP-1 members enhances target gene activation and cellular growth.

**Significance:** c-Jun-dependent cooperativity between TAp73 and selected AP-1 members contributes to cellular growth.

## Introduction

Members of the p53 tumor suppressor family including p63 and p73 are crucial for the maintenance of balance between cell survival and death in physiological and pathological conditions (1–3). They are thus activated by both stress signals and growth factors, suggesting pleiotropic roles in a multitude of cellular processes (4–6). Being transcription factors, transactivation of relevant target genes has been shown to be essential for their functions (7, 8). Consistently, in a high percentage of cancers, p53 is mutated in the DNA-binding domain, thereby impairing its transactivation function (9). However, p73 is rarely mutated but is in fact overexpressed in cancers (10, 11), highlighting potentially disparate functions among these similar proteins. Interestingly, p73-null mice do not develop tumors at an accelerated rate as do the p53-null mice (12, 13) and instead show several developmental defects of the central nervous system, thus indicating that p73 has evolved to perform functions beyond tumor suppression (14).

p73 exists in two major forms. The first major form is the full-length TAp73, which is structurally and functionally homologous to p53 but appears also to possess several properties distinct from p53 that can be associated with different promoter specificity of the two related proteins (7, 8, 15). The presence of the second internal promoter leads to the generation of the second major form, the ΔNp73 (16). The latter lacks the amino-terminal transactivation domain and hence acts as a dominant negative inhibitor of both TAp73 and p53, giving rise to the notion that TAp73 is a tumor suppressor like p53, whereas ΔNp73 is an oncogene (17). Recent generation of isoform-specific knock-out mice has been instrumental in clarifying some of the roles of TAp73 and ΔNp73 (13, 18). Absence of ΔNp73 was shown to lead to neurodegeneration and to sensitize cells to DNA damage-inducing agents, suggesting that it might play a role in chemoresistance (18). Conversely, absence of TAp73 leads to infertility and hippocampal dysgenesis (13). Loss of TAp73 also leads to genomic instability, although this was suggested to occur in a cell type-specific manner, indicating that cellular context can dictate the biological outcome of p73 activity.

Although the overexpression of ΔNp73 in several cancers is expected, significant data also exist demonstrating the overexpression of TAp73 in various cancers (19–21). Importantly, several lines of evidence indicate that TAp73 expression can be associated with promoting cellular survival and antiapoptosis under certain conditions. For example, we have previously shown that TAp73 can induce the expression of the antiapoptotic caspase 2S, leading to resistance to cell death in neuroblastoma cells (22). Also, expression of TAp73 in conjunction with p53 can suppress p53-mediated telomerase activation via activation of HDM2 (23). TAp73 was also found to bind to p53-responsive elements in promoters of cell cycle progression genes, causing aberrant activation of cell proliferation (24), and recently, TAp73 was shown to regulate the pentose phosphate pathway to promote cellular proliferation (25). Finally, several proto-oncogenes have also been shown to induce and activate TAp73 isoforms (5). One such proto-oncogene is the activator protein (AP)^2^-1 family member c-Jun, which has been shown to stabilize TAp73 (26).

The mammalian AP-1 proteins belong to the Jun (c-Jun, JunB, and JunD), Fos (c-Fos, FosB, Fra1, and Fra2), and closely related activating factor (ATF-2, ATF-3, and B-ATF) families. These proteins can homo- or heterodimerize and can regulate embryonic development, cell survival, or death by means of inducing and/or repressing tumor suppressor genes or cell cycle regulatory genes (27, 28). For instance, c-Jun is required for proliferation of fibroblasts and other cultured cells (29) and has been demonstrated to also inhibit p53-induced growth arrest upon ultraviolet (UV) irradiation (30). In contrast, dominant negative c-Jun mutant could protect sympathetic neurons against NGF withdrawal-induced apoptosis (31). Moreover, accumulating evidence points to differing combinations of AP-1 dimers having different biological functions as has been suggested in the antagonistic regulation of *peroxisome proliferator-activated receptor* γ, thereby affecting obesity and hepatic functions (32). Thus, the cell type- and context-dependent signals could alter AP-1 dimer composition and thus dictate the eventual cellular fate.

Previously, we have shown that co-expression of TAp73 with c-Jun stabilizes TAp73 and leads to increased cellular survival via the up-regulation of cyclin D1 at both the RNA and protein levels (26, 33). This up-regulation is dependent on c-Jun and is required for TAp73-induced cell proliferation. Furthermore, induction of another AP-1 target gene (collagenase/MMP-1) upon treatment with the tumor-promoting phorbol ester (12-*O*-tetradecanoylphorbol-13-acetate) was impaired in p73-null mouse embryonic fibroblasts (MEFs). These data suggested that TAp73 plays a vital role in activation of endogenous AP-1 target genes, thereby contributing to cellular survival. To further understand the details of how TAp73 acts as a growth promoter, we analyzed the mechanistic basis of AP-1 target gene activation by TAp73 and the composition of AP-1 family members involved in this process.

## Experimental Procedures

### 

#### 

##### Cell Culture, Plasmids, and Transfections

The p53-null human lung cancer cell lines H1299 (parental and with doxycycline-inducible TAp73), the osteosarcoma cell line Saos-2 (parental and with doxycycline-inducible TAp73), and mouse embryonic fibroblasts lacking p53 (p53^−/−^) or p53 and c-Jun (p53^−/−^c-Jun^−/−^) were used. Cells were grown in DMEM supplemented with 10% bovine fetal serum (Gibco). The inducible cells were maintained in DMEM supplemented with 10% tetracycline-free serum (Invitrogen). Cells were induced with doxycycline (Sigma; 2 μg/ml).

Luciferase reporter plasmids used include the following: collagenase promoter, minimal collagenase (ΔCol) promoter, cyclin D1 promoter with or without deletion of the 12-*O*-tetradecanoylphorbol-13-acetate-responsive element (TRE/AP-1 site) and cyclic AMP-responsive element (CRE) binding sites (34, 35). AP-1 site mutants of the collagenase promoter were generated by site-directed mutagenesis using the following primers: collagenase promoter-proximal AP-1 site mutation, 5′-GAAAGCCAGAGGCTGTCTACCTCATCAAGCTTGGATC-3′ and 5′-GATCCAAGCTTGATGAGGTAGACAGCCTCTGGCTTTC-3′; collagenase promoter-distal AP-1 site mutation, 5′-GGCAATCATTAGAAATGGTACCTCCTAGCAGATTATTTGG-3′ and 5′-CCAAATAATCTGCTAGGAGGTACCATTTCTAATGATTGCC-3′; ΔCol promoter AP-1 site mutation, 5′-GAAAGCCAGAGGCTGTCTACCTCATGCTTTATAACATC-3′ and 5′-GATGTTATAAAGCATGAGGTAGACAGCCTCTGGCTTTC-3′. Expression vectors (all in pcDNA3) expressing p53, TAp73β, ΔNp73β, TAp73^R292H^, deletion mutants of TAp73, c-Jun, and other AP-1 family members have been described previously (23, 26, 36, 37). The c-Jun^AA^ mutant was generated from FLAG-tagged c-Jun by site-directed mutagenesis using the following primers; S63A primers, 5′-GACCTCCTCACCGCGCCCGACGTGG-3′ and 5′-CCACGTCGGGCGCGGTGAGGAGGTC-3′; S73A primers, 5′-CTCAAGCTGGCGGCGCCCGAGCTGG-3′ and 5′-CCAGCTCGGGCGCCGCCAGCTTGAG-3′. Other c-Jun mutant cDNAs were subcloned into 3xCMV FLAG vector from already described expression vectors (38, 39). Plasmids were transfected using Lipofectamine Plus reagent (Invitrogen) according to the manufacturer's protocol. The total amount of transfected DNA was equalized with appropriate amounts of pcDNA3 vector in all cases.

For modified chromatin immunoprecipitation (ChIP-IP), transfected cells were incubated in serum-free medium overnight for serum starvation, and 20% serum medium was added for 4 h before harvesting. Transfected cells were also incubated in normal medium overnight and exposed to UV irradiation (60 J) for 4 h before harvesting.

##### siRNA and Transfections

siRNAs for human c-Jun and Fra1 were purchased from Santa Cruz Biotechnology (sc-29223 and sc-35405). siRNAs for control (scrambled), human c-Fos, and human Fra2 (TTCTCCGAACGTGTCACGT, AGGAGAATCCGAAGGGAAA, and GCGCTGTAGTGGTGAAACA, respectively) were synthesized. siRNAs were transfected using Transmessenger (Qiagen) following the manufacturer's protocol. 24 h after siRNA transfection, the indicated plasmids were transfected using Lipofectamine Plus reagent as mentioned above.

##### RNA Analysis

Total RNA was prepared from cells using TRIzol reagent (Invitrogen) according to the manufacturer's instructions. 1.5–3 μg of total RNA was reverse transcribed into cDNA using Superscript II (Invitrogen). Semiquantitative reverse transcription-PCR analysis was performed using the following primers: c-Fos, 5′-CCAACCTGCTGAAGGAGAAG-3′ and 5′-GCTGCTGATGCTCTTGACAG-3′; Fra2, 5′-GAGTTCATGTTGGTGGCTCA-3′ and 5′-TTCTGCGGTGAGCCTTGGA-3′.

##### Luciferase Assays

H1299 cells were seeded in 6-well plates and transiently transfected with appropriate plasmids (0.3–0.5 μg) and β-galactosidase gene (50 ng) for normalization. Cells were washed and lysed in luciferase lysis buffer 24 h post-transfection, and luciferase assays were performed as described (23, 26).

##### Immunoprecipitation and Immunoblot Analysis

Cell lysates were prepared in lysis buffer containing 0.5% Nonidet P-40 as described (36). For immunoprecipitation, 0.5–1.0 mg of lysate was used with agarose-immobilized anti-FLAG M2 antibody (Stratagene). Bead-bound proteins were isolated by boiling in 2× SDS sample buffer followed by separation on SDS-polyacrylamide gels. Immunoblotting was performed with the following antibodies: anti-p73 (ER15 and GC15, Oncogene), anti-FLAG M2, anti-phosphorylated c-Jun Ser-63 (9261, Cell Signaling Technology), anti-c-Jun (60A8, Cell Signaling Technology), anti-Fra1 (R-20, Santa Cruz Biotechnology), and anti-actin (Sigma).

##### Chromatin Immunoprecipitation (ChIP) and ChIP-IP

ChIP assays were carried out as described previously (33). The following primer sets were used for analysis of the transcription factor binding on the target gene promoters: Mdm2, 5′-GATCGCAGGTGCCTGTCGGGTCACTA-3′ and 5′-GGTCTACCCTCCAATCGCCACTGAACACA-3′; cyclin A1, 5′-CTCTTAACCGCGATCCTCCAG-3′ and 5′-CAATAAAAGATCCAGGGTACATGATTG-3′; cyclin D1, 5′-TCAGAGGTGTGTTTCTCCCGGTTAAATTG-3′ and 5′-GGTGGCCAGCATTTCCTTCATCTTGT-3′; cyclin D1 neg, 5′-CTGGCCATGAACTACCTGGA-3′ and 5′-GTCACACTTGATCACTCTGG-3′.

The ChIP protocol was modified as follows for ChIP-IP. 24 h after induction or transfection, cells (10-cm plate) were fixed with 1% formaldehyde for 10 min at room temperature. Cells were then washed twice with ice-cold PBS and lysed for 20 min using 4 ml of 0.25% Triton X-100 lysis buffer (0.25% Triton X-100, 20 mm EDTA, 10 mm Tris-HCl, pH 8.1) with protease inhibitors at 4 °C. Samples were centrifuged at 750 × *g* at 4 °C for 5 min. The pellets were lysed in 400 μl of 1% SDS lysis buffer (1% SDS, 10 mm EDTA, 50 mm Tris-HCl, pH 8.1) with protease inhibitors, and the extracts were sonicated on ice with 10-s pulses for 4 min each. Samples were centrifuged at 13,000 rpm at 4 °C for 30 min, and supernatant was collected. Samples were diluted to 2 ml in ChIP dilution buffer (1% Triton X-100, 2 mm EDTA, 20 mm Tris-HCl, pH 8, 150 mm NaCl) with protease inhibitors. 40 μl of the diluted sample was kept aside as the input fraction before preclearing with mouse IgG-agarose beads (10 μl/sample) for 4 h at 4 °C. The supernatant was incubated with anti-FLAG-agarose beads (15 μl/sample) (M2 affinity gel, Sigma) for 2 h at 4 °C. Immune complexes were washed one time with LS buffer (0.1% SDS, 1% Triton X-100, 2 mm EDTA, 20 mm Tris-HCl, pH 8, 150 mm NaCl), one time with HS buffer (0.1% SDS, 1% Triton X-100, 2 mm EDTA, 20 mm Tris-HCl, pH 8, 500 mm NaCl), one time with 0.25 m LiCl buffer (250 mm LiCl, 1% deoxycholate, 1 mm EDTA, 1% Nonidet P-40, 10 mm Tris-HCl, pH 8), and two times with Tris-EDTA buffer. DNA·protein complex was eluted two times with 40 μl of elution buffer (1% SDS, 0.1 m NaHCO_3_). Cross-links were reversed by addition of 200 mm NaCl at 65 °C for 4 h followed by DNase I treatment (7.5 Kunitz units/sample; Qiagen) at 37 °C for 2 h. Protein concentration was measured, and samples were lysed in SDS sample buffer.

##### Colony Formation Assays

For colony formation assays, H1299 cells were transfected with the indicated plasmids and grown in appropriate selection medium (G418) for between 10 and 14 days. Surviving colonies were stained with crystal violet solution (Merck) as described previously (33). Quantifications were done using MetaMorph Offline (Version 7.8.0; Molecular Devices, LLC), which quantifies live cells by percent colony area.

##### Statistical Analysis

Data were analyzed by two-way analysis of variance. The differences in mean values were considered significant at *p* values ≤0.001 (***), ≤0.01 (**), and ≤0.05 (*).

## Results

### 

#### 

##### Some Fos Family Members Cooperate with TAp73 to Potentiate AP-1 Target Gene Activation

**FIGURE 1.**
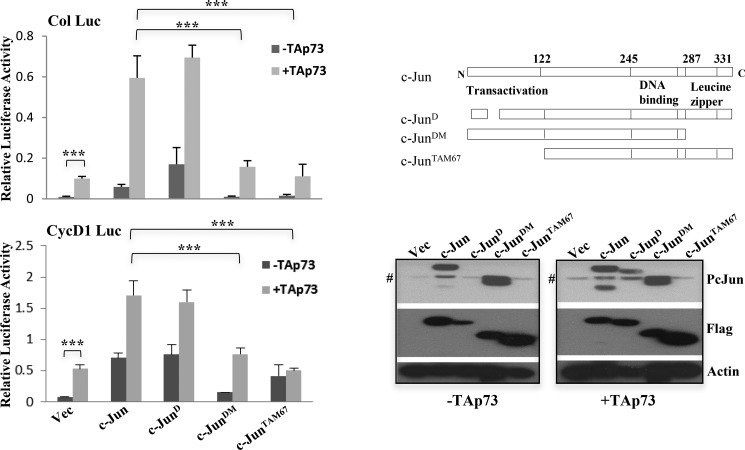
**Dimerization and transactivation domains of c-Jun are required for cooperation with TAp73 to activate AP-1 target gene transactivation.** Activation of human collagenase promoter-luciferase (*Col Luc*) and cyclin D1 promoter-luciferase (*CycD1 Luc*) constructs was determined by luciferase activity in p53-null H1299 cells transfected with the different c-Jun constructs either in the absence or presence of TAp73β. The schematic on the *top right* shows the c-Jun deletion mutants used (c-Jun^DM^, dimerization mutant; c-Jun^D^, delta domain mutant; c-Jun^TAM67^, transactivation mutant). All the c-Jun constructs are FLAG-tagged. The Western blot shows the expression levels of the different c-Jun constructs from the corresponding luciferase assay lysates. *Pc-Jun* indicates expression of the Ser-63-phosphorylated form of c-Jun. # indicates a nonspecific band. All luciferase experiments were repeated three to five times. Data show the mean of experiments, and *error bars* indicate S.D. ***, *p* ≤ 0.001 comparing WT c-Jun with the various mutants in combination with TAp73β. *Vec*, vector.

We have shown previously that TAp73 is capable of transactivating AP-1 target genes such as cyclin D1 and collagenase in a manner dependent on the expression of c-Jun (33). Moreover, TAp73 was able to synergize with c-Jun to further potentiate the activation of AP-1 target genes (Fig. 1) unlike p53 (33). To gain further mechanistic insights into TAp73-mediated AP-1 activation, we first evaluated the role of the functional domains of c-Jun that are required for cooperation with TAp73. c-Jun deletion mutants that lack the leucine zipper domain that is required for homo- and heterodimerization (c-Jun^DM^), the delta domain that is required for docking of Jun N-terminal kinases (c-Jun^D^), or the N-terminal transactivation domain (c-Jun^TAM67^) were used. The schematic shows the regions deleted, and the Western blot shows expression of all the c-Jun mutant constructs (all in 3xFLAG CMV vector). Unlike wild-type c-Jun, c-Jun^DM^ or c-Jun^TAM67^ was unable to cooperate with TAp73 to activate both the human collagenase and the cyclin D1 promoter-luciferase constructs (Fig. 1). In contrast, c-Jun^D^ mutant alone was able to activate both promoters even though phosphorylation at serine 63 was not detected, suggesting that JNK-mediated phosphorylation is not important. Additionally, TAp73 was able to cooperate with the c-Jun^D^ mutant, suggesting that activation by JNK was not a prerequisite for the cooperativity, consistent with our previous findings that TAp73 is capable of cooperating with c-Jun in a JNK-independent manner (33). Furthermore, co-expression with TAp73 led to the phosphorylation of the c-Jun^D^ mutant, confirming JNK-independent phosphorylation of c-Jun in the presence of TAp73 as suggested earlier (33).

**FIGURE 2.**
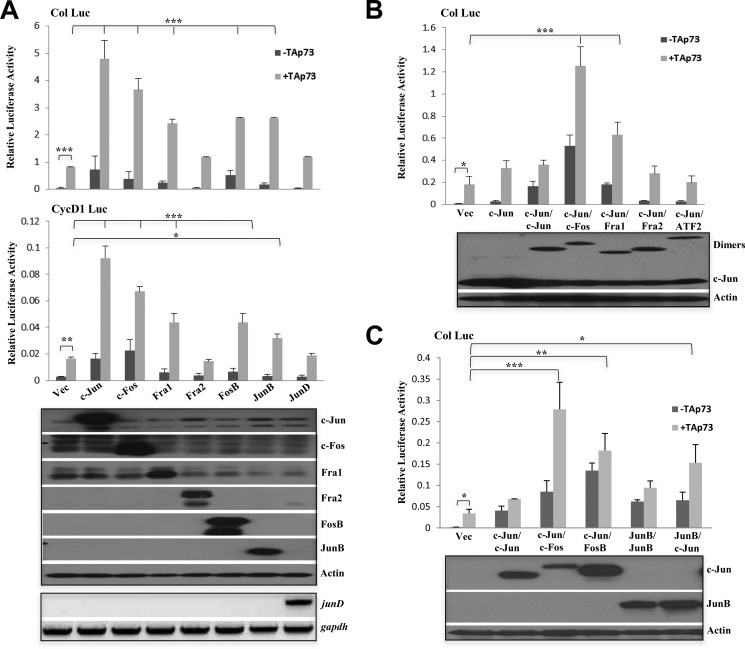
**Some Fos family members are required for cooperation with TAp73.** *A*, activation of collagenase promoter-luciferase (*Col Luc*) (*upper panel*) and cyclin D1 promoter-luciferase (*CycD1 Luc*) (*lower panel*) in H1299 cells transfected with the different AP-1 members either in the absence or presence of TAp73β. The Western blot shows the expression of transfected AP-1 members in the luciferase lysates. *junD* expression was analyzed by RT-PCR (*lower panel*). *B* and *C*, activation of collagenase promoter-luciferase in H1299 cells transfected with the various single chain AP-1 dimers either in the absence or presence of TAp73β. The Western blot below depicts expression levels of the single chain dimers. All luciferase experiments were repeated three to five times. Data show the mean of experiments, and *error bars* indicate S.D. ***, *p* ≤ 0.001; **, *p* ≤ 0.01; *, *p* ≤ 0.05 comparing c-Jun with other AP-1 members (*A*) or the dimers (*B* and *C*) in combination with TAp73β. *Vec*, vector.

Although the inability of the c-Jun^TAM67^ to cooperate with TAp73 was expected due to the lack of the transactivation domain, the defect due to the c-Jun^DM^ mutant suggested the requirement of c-Jun dimerization for cooperation with TAp73. Because the dimerization domain is an important component for c-Jun to homo- or heterodimerize with its family members, we assessed the role of other AP-1 family members to cooperate with TAp73. We found that like c-Jun, expression of c-Fos could synergize with TAp73 to activate the collagenase promoter-luciferase construct to significant levels (Fig. 2*A*). Other than c-Fos, Fra1, FosB, and JunB could also cooperate with TAp73 albeit to lesser but significant levels (Fig. 2*A*, *upper panel*). Fra2 and JunD were unable to cooperate at all with TAp73. Similar results were obtained with cyclin D1 promoter-luciferase construct (Fig. 2*A*, *lower panel*). This suggested selectivity among AP-1 family members in their ability to cooperate with TAp73 to activate specific target genes. To determine whether specific AP-1 dimer pairs are therefore better able to cooperate with TAp73, we evaluated the effects of some specific single chain AP-1 dimers, which have been used to demonstrate AP-1 member-pairing specificity (37, 40). Surprisingly, although c-Jun homodimers did not cooperate as well with TAp73, the c-Jun/c-Fos and c-Jun/Fra1 dimers showed a high level of synergy in activating the collagenase promoter-luciferase construct (Fig. 2*B*). Like c-Jun homodimers, c-Jun/Fra2 and c-Jun/ATF2 heterodimers did not synergize well with TAp73. Because JunB and FosB could also cooperate with TAp73 (Fig. 2*A*), we further assessed whether c-Jun/FosB, JunB/JunB, or JunB/c-Jun dimers could synergize with TAp73 (Fig. 2*C*). c-Jun/FosB dimers were capable of activating the collagenase promoter-luciferase construct and cooperated marginally with TAp73. However, similar to c-Jun homodimers, JunB homodimers could not cooperate with TAp73, whereas JunB/c-Jun cooperated with TAp73 although less efficiently as compared with c-Jun/c-Fos dimers probably due to promoter specificity of the AP-1 members as has been described (32, 40). These data, therefore, suggest that heterodimerization of c-Jun with certain AP-1 family members, specifically the Fos family members (c-Fos, Fra1, and FosB but not Fra2), is important for cooperation with TAp73 to mediate AP-1 target gene transactivation.

##### c-Fos and Fra1 Are Required for TAp73-mediated AP-1 Target Gene Activation in a c-Jun-dependent Manner

**FIGURE 3.**
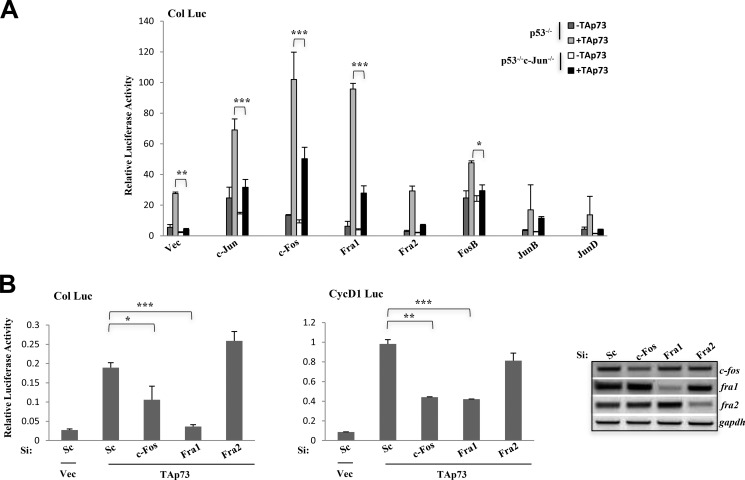
**c-Fos and Fra1 are required in a c-Jun-dependent manner to regulate TAp73-mediated transactivation of AP-1 targets.** *A* and *B*, activation of collagenase promoter-luciferase (*Col Luc*) by different AP-1 members either in the absence or presence of TAp73β in p53^−/−^ and p53^−/−^c-Jun^−/−^ MEFs (*A*) or of collagenase promoter-luciferase and cyclin D1 promoter-luciferase (*CycD1 Luc*) by TAp73β in H1299 cells either in the absence or presence of scrambled (*Sc*), c-Fos, Fra1, or Fra2 siRNA (*Si*) (*B*). RT-PCR shows the extent of knockdown. All luciferase experiments were repeated three to five times. Data show the mean of experiments, and e*rror bars* indicate S.D. ***, *p* ≤ 0.001; **, *p* ≤ 0.01; *, *p* ≤ 0.05 comparing c-Jun in combination with TAp73β in p53^−/−^ *versus* p53^−/−^c-Jun^−/−^ MEFs (*A*) and scrambled siRNA with AP-1-specific siRNAs (*B*). *Vec*, vector.

We next assessed whether the strong cooperation of the other AP-1 members with TAp73 is dependent on c-Jun. Therefore, we overexpressed c-Fos, Fra1, and other AP-1 members and assessed the synergistic effects with TAp73 on the collagenase promoter-luciferase construct in p53^−/−^c-Jun^−/−^ and p53^−/−^ MEFs (Fig. 3*A*). TAp73 strongly synergized with c-Jun, c-Fos, and Fra1 in p53^−/−^ MEFs, suggesting that this cooperation can be broadly recapitulated independently of the cell type used, although there were subtle differences as in the case with FosB, which did not show as robust a cooperation, and JunB, which did not synergize at all. Nonetheless, Fra2 and JunD did not cooperate with TAp73 as noted earlier. Interestingly, the synergistic effect between TAp73 and c-Fos/Fra1 was decreased significantly in p53^−/−^c-Jun^−/−^ MEFs, indicating that the cooperation of TAp73 with c-Fos and Fra1 was dependent on c-Jun. Even though FosB did not synergize robustly with TAp73 in the murine cell line, there was a decrease in cooperation in the p53^−/−^c-Jun^−/−^ MEFs, again indicating dependence on c-Jun. However, the residual activation of the collagenase promoter-luciferase construct by c-Fos and Fra1 in p53^−/−^c-Jun^−/−^ MEFs indicates potential compensation by other Jun members. Similar results were obtained with cyclin D1 promoter-luciferase construct (data not shown). Because c-Fos and Fra1 cooperated with TAp73 in a c-Jun-dependent manner consistently in both human and murine cell lines, we further investigated whether abrogation of c-Fos or Fra1 expression would affect TAp73-mediated AP-1 target gene activation. siRNA-mediated knockdown of c-Fos or Fra1 expression indeed significantly reduced TAp73-mediated activation of collagenase and cyclin D1 promoter-luciferase activities, whereas knockdown of Fra2 expression did not have any effect (Fig. 3*B*). Together, the data indicate that TAp73 cooperates with specific AP-1 family members like c-Fos and Fra1 in a c-Jun-dependent manner to mediate AP-1 target gene activation.

##### TRE/AP-1 Site Is Required for the Efficient Transactivation of AP-1 Promoters by TAp73

**FIGURE 4.**
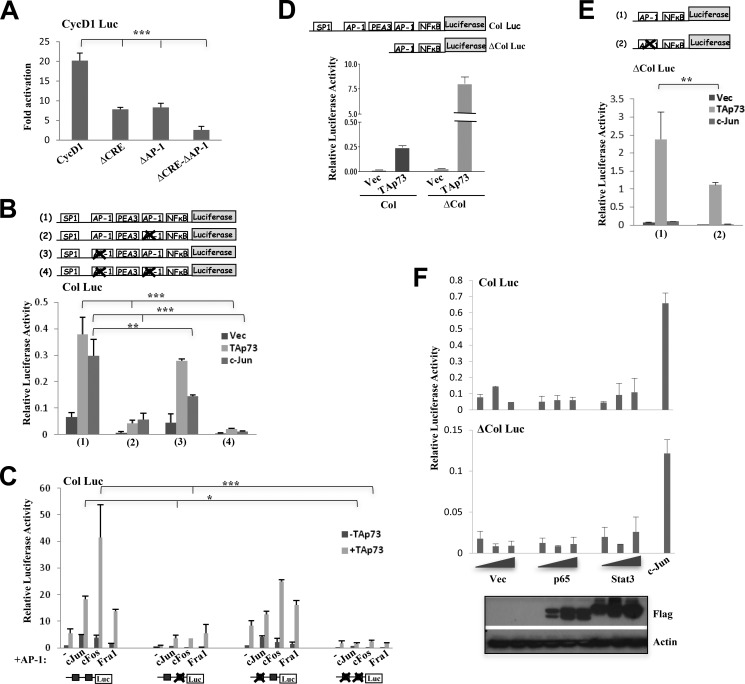
**TAp73 requires the TRE/AP-1 site for efficient activation of AP-1 target gene promoters.** *A*, activation of the cyclin D1 promoter-luciferase (*CycD1 Luc*) or the mutants thereof including the ΔCRE in which the cAMP response element is deleted, the ΔAP-1 in which the AP-1 site is deleted, or the construct in which both sites are deleted (ΔCRE-ΔAP1) by TAp73β was analyzed after transfection in H1299 cells. *B*, activation of collagenase promoter-luciferase (*Col Luc*) and AP-1 site mutants thereof by TAp73β or c-Jun (*lower panel*). The *upper panel* shows schematics of the full-length collagenase promoter-luciferase construct containing two AP-1 sites (*1*) and constructs in which the proximal (*2*), distal (*3*), or both (*4*) AP-1 sites were mutated. *C*, cooperation between TAp73β and the AP-1 members in the activation of the various collagenase promoter-luciferase promoter constructs in H1299 cells transfected with the different AP-1 members in the absence or presence TAp73β. *D* and *E*, activation of collagenase promoter-luciferase (*Col Luc*) with two AP-1 sites, the ΔCol Luc with one AP-1 site (*D*), or the ΔCol Luc in which the AP-1 site was mutated (*E*) by TAp73β or c-Jun in H1299 cells. Schematics show the ΔCol Luc construct without or with the mutated AP-1 site. *F*, activation of collagenase promoter-luciferase (*Col Luc*) and ΔCol Luc in H1299 cells transfected with increasing concentrations (0.1, 0.4, and 0.75 μg) of STAT3 and p65 expression plasmids. c-Jun was transfected as a positive control. The Western blot shows the expression level of STAT3 and p65. All luciferase experiments were repeated three to five times. Data show the mean of experiments, and *error bars* indicate S.D. ***, *p* ≤ 0.001; **, *p* ≤ 0.01; *, *p* ≤ 0.05 comparing the effect of TAp73β on WT with mutant cyclin D1 promoter constructs (*A*) or the effect of TAp73β and/or c-Jun on collagenase promoter (*B* and *C*) and ΔCol Luc constructs (*E*). *Vec*, vector.

TAp73 requires AP-1 proteins such as c-Jun, c-Fos, and Fra1 and their dimerization for transactivation of AP-1 targets, suggesting that this phenomenon occurs via the AP-1 binding site on the promoters. Among the AP-1 gene promoters used, human cyclin D1 promoter has one AP-1 site, whereas human collagenase promoter has two AP-1 sites that are required for both constitutive and inducible expression (41–43). We found that TAp73 could only partially activate the cyclin D1 promoter lacking the AP-1 site (ΔAP-1), indicating that the AP-1 site is important for this process (Fig. 4*A*). However, deletion of the CRE (ΔCRE) also partially inhibited TAp73-mediated activation, indicating that TAp73 can act via other elements or signaling pathways. Consistently, deletion of both the AP-1 site and the CRE (ΔAP-1-ΔCRE) completely abrogated TAp73-mediated activation of the cyclin D1 promoter. To further confirm the requirement of AP-1 site, we analyzed the two AP-1 sites in the human collagenase promoter. To determine whether either of the AP-1 sites are necessary for TAp73 activity, either the promoter-proximal AP-1 site (denoted as 2) or the promoter-distal AP-1 site (denoted as 3) were mutated (Fig. 4*B*). Mutation of the promoter-proximal AP-1 site completely abrogated activation of the promoter by both c-Jun and p73. However, mutation of the promoter-distal AP-1 site only partially inhibited activation by c-Jun and TAp73. This suggested that the promoter-proximal AP-1 site in the collagenase promoter is required for activation by both c-Jun and TAp73 and that not all AP-1 sites are utilized in the same manner by these transcription factors. Mutation of both AP-1 sites completely abolished both c-Jun- and TAp73-mediated activation. As expected, the promoter-proximal AP-1 site in the human collagenase promoter is also important in cooperation with c-Jun and other AP-1 members as mutation of this site almost completely inhibited synergistic activation by TAp73 and the AP-1 proteins (c-Jun, c-Fos, and Fra1) (Fig. 4*C*).

Because the promoter-proximal AP-1 site was more predominantly utilized by TAp73, we tested the ΔCol promoter that mainly harbors only the promoter-proximal AP-1 site besides the NFκB and STAT3 transcription factor binding sites. Activation of the minimal promoter was at least 10-fold higher than the full-length collagenase promoter, confirming that TAp73 can strongly activate the collagenase promoter through this AP-1 site (Fig. 4*D*). Furthermore, strong activation of the minimal promoter by TAp73 suggests that there may be certain repressive elements in the full-length promoter. Mutation of the AP-1 site on the minimal promoter completely abrogated activation by c-Jun and significantly inhibited activation by TAp73, suggesting that although the AP-1 family members are crucial other factors may play a role in TAp73-mediated activation (Fig. 4*E*). This correlates well with the results obtained from the cyclin D1 promoter mutants (Fig. 4*A*). To evaluate this possibility, we tested whether the other response elements in the minimal promoter (*i.e.* NFκB/p65 and STAT3) could be activated by the appropriate transcription factors and whether they could synergize with TAp73. However, neither STAT3 nor p65 were found to activate the full-length or minimal collagenase promoter (Fig. 4*F*), and neither synergized with TAp73 (data not shown). These data together demonstrate the importance of the AP-1 binding sites on the target promoters for transactivation by TAp73 with AP-1 family members.

##### TAp73 Binds at or Near the AP-1 Site to Activate Target Genes

**FIGURE 5.**
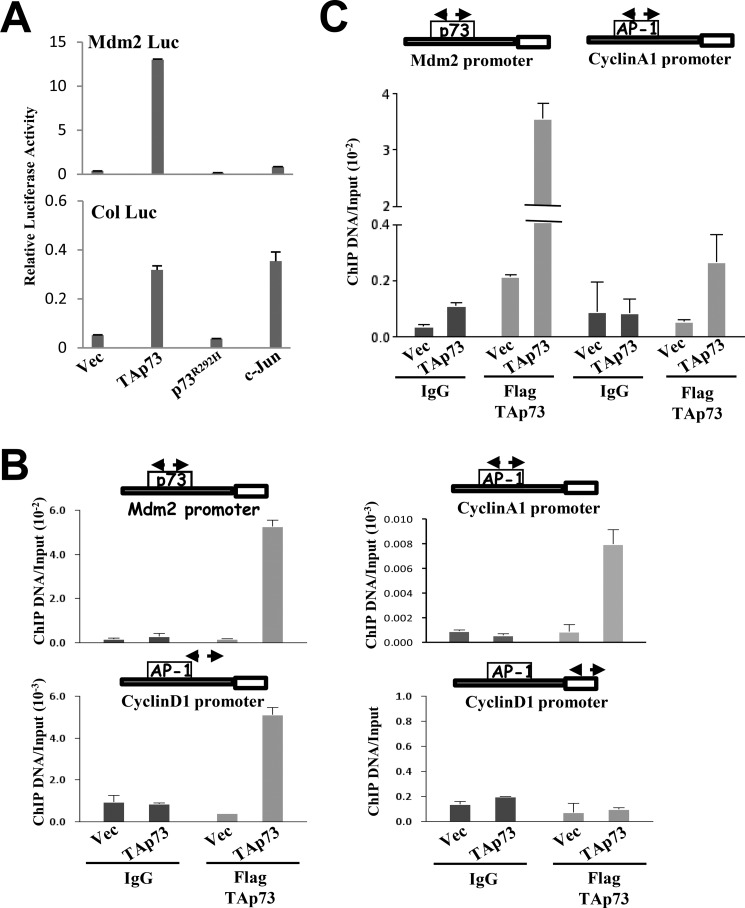
**TAp73 binds to AP-1 target gene promoter elements.** *A*, activation of human *mdm2* promoter-luciferase (*Mdm2 Luc*) and collagenase promoter-luciferase (*Col Luc*) in H1299 cells transfected with the various TAp73β forms and c-Jun. *B*, H1299 cells were transfected with either CMV-FLAG empty vector or the CMV-FLAG-TAp73β plasmid and subjected to ChIP with control IgG or anti-FLAG antibodies. TAp73β binding to its canonical site on the human *mdm2* promoter (*top left*), at the AP-1 site on the human cyclin A1 promoter (*top right*), or near the AP-1 site on the human cyclin D1 promoter (*bottom left*) was analyzed by real time PCR. The regions amplified are shown in the schematics in the *insets*. Binding of TAp73β to an internal region of cyclin D1 gene away from the AP-1 site was also analyzed (*bottom right*) as a negative control. The ChIP experiments were repeated at least three times independently in duplicates, and representative results are shown. *Error bars* indicate S.D. between duplicates. *C*, comparative analysis of relative binding of TAp73β to the *mdm2* promoter and the cyclin A1 promoter determined by semiquantitative PCR analysis in the same experiment. *Vec*, vector.

TAp73 has been shown to bind to the promoter of its canonical target genes such as *mdm2* to transactivate their expression. However, our previous gel shift studies indicated that TAp73 was unable to bind to the exact short canonical TRE sites of AP-1 target genes (33). Nonetheless, given that TAp73 was able to cooperate with AP-1 members in a TRE-dependent manner, we re-evaluated whether the DNA binding capacity of TAp73 is required for this process by using a DNA-binding domain-defective mutant, TAp73^R292H^ (44). As expected, TAp73^R292H^ was defective in the transactivation of the *mdm2* promoter (Fig. 5*A*). However and surprisingly, this mutant was also defective in the activation of the collagenase promoter (Fig. 5*A*), thus indicating that DNA binding is a prerequisite for AP-1 target gene activation. Therefore, we explored whether TAp73 can directly bind to the AP-1 target gene promoters at or near the canonical AP-1 site. Chromatin immunoprecipitation assays in cells transfected with TAp73 showed that TAp73 binds at or near the AP-1 sites in cyclin D1 and cyclin A1 regulatory regions (Fig. 5*B*) but not to an unrelated site as assayed with an internal region in the cyclin D1 gene (Fig. 5*B*). However, side-by-side comparison revealed that the binding of TAp73 to these sites on AP-1 target genes is much weaker compared with its binding efficiency at its canonical binding site on the *mdm2* promoter (Fig. 5*C*). Nonetheless, these data highlight the ability of TAp73 to bind to DNA for the transactivation of AP-1 target genes.

##### TAp73 Interacts with c-Jun at the Chromatin

**FIGURE 6.**
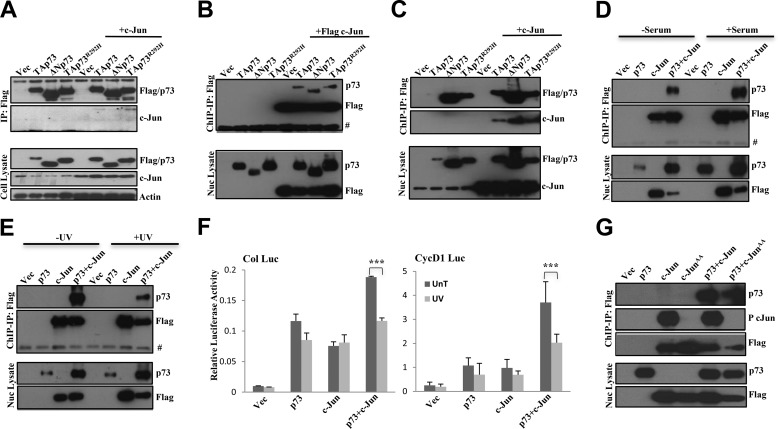
**p73 interacts with c-Jun on the chromatin.** *A*, H1299 cells were transfected with FLAG-tagged TAp73β, ΔNp73β, or TAp73β^R292H^ in the presence or absence of c-Jun, and whole cell lysates were used for IPs with anti-FLAG antibody beads followed by immunoblotting with the indicated antibodies (*upper panels*). *B*, H1299 cells were transfected with TAp73β, ΔNp73β, or TAp73β^R292H^ in the presence or absence of FLAG-tagged c-Jun, and nuclear (*Nuc*) lysates were used for the modified ChIP protocol as mentioned under “Experimental Procedures” for IP with anti-FLAG antibody followed by immunoblotting with the indicated antibodies (*upper panels*). # indicates a nonspecific band. *C*, H1299 cells were transfected with FLAG-tagged TAp73β, ΔNp73β, or TAp73β^R292H^ in the presence or absence of c-Jun, and nuclear lysates were used for the modified ChIP for IP with anti-FLAG antibody followed by immunoblotting with the indicated antibodies (*upper panels*). *D*, H1299 cells were transfected with TAp73β in the presence or absence of FLAG-tagged c-Jun, left in serum-free medium overnight and fed with 20% serum-containing medium (+*serum*) or otherwise (−*serum*) for 4 h, and nuclear lysates were collected and used for the modified ChIP for IP with anti-FLAG antibody followed by immunoblotting with the indicated antibodies (*upper panels*). # indicates a nonspecific band. *E* and *F*, H1299 cells were transfected with TAp73β in the presence or absence of FLAG-tagged c-Jun, left untreated or treated with UV irradiation (60 J). Nuclear lysates were collected 4 h later and used for the modified ChIP for IP with anti-FLAG antibody followed by immunoblotting with the indicated antibodies (*upper panels*) (*E*). # indicates a nonspecific band. Activation of collagenase promoter-luciferase (*Col Luc*) and cyclin D1 promoter-luciferase (*CycD1 Luc*) in the untreated (*UnT*) or UV-treated condition in concurrent experiments is shown (*F*). Data show the mean of three to five experiments, and *error bars* indicate S.D. ***, *p* ≤ 0.001 comparing c-Jun in combination with TAp73β in untreated *versus* UV-treated condition. *G*, H1299 cells were transfected with TAp73β in the presence or absence of FLAG-tagged c-Jun or c-Jun^AA^ mutant, and nuclear lysates were used for the modified ChIP with anti-FLAG antibody followed by immunoblotting with the indicated antibodies (*upper panels*). The *lower panels* show straight immunoblot data from the lysates without IP in all cases. Experiments were repeated at least two times, and representative results are shown. *Vec*, vector.

To better understand the synergy between TAp73 and c-Jun, we examined the interaction between them given that TAp73 is able to bind to AP-1 target gene regulatory regions. Cells were transfected with FLAG-tagged c-Jun and various forms of untagged TAp73 (*i.e.* transactivation domain-deleted ΔNp73 and TAp73^R292H^). As reported previously (26), we were unable to observe binding between the various p73 forms and c-Jun in co-immunoprecipitation experiments by immunoprecipitating c-Jun (data not shown) or in reverse co-immunoprecipitation experiments where FLAG-tagged p73 forms were immunoprecipitated (Fig. 6*A*), indicating that TAp73 and c-Jun do not interact under normal conditions in the soluble cellular fractions. Nonetheless, because both c-Jun and TAp73 bind to or near the AP-1 site on the target promoters, we hypothesized that c-Jun and TAp73 may interact at the level of chromatin. To test this, we isolated chromatin-bound FLAG-tagged c-Jun from transfected cells using a modified chromatin immunoprecipitation protocol and found that c-Jun can indeed interact with TAp73, ΔNp73, and even the DNA binding mutant TAp73^R292H^ (Fig. 6*B*). Reverse immunoprecipitation experiments using chromatin-bound FLAG-tagged p73 forms confirmed that this interaction indeed occurred at the level of chromatin (Fig. 6*C*) regardless of the absence of the transactivation domain or the ability to bind to DNA.

Both TAp73 and c-Jun are activated and up-regulated during both growth factor stimulation and genotoxic stress (5, 6). Therefore, we assessed the status of interaction between TAp73 and c-Jun during stress conditions such as UV irradiation as well as during prosurvival condition-associated growth factor signaling. Chromatin-bound FLAG-tagged c-Jun was isolated from transfected cells, and co-immunoprecipitation revealed the association with TAp73 both under serum-starved or serum-stimulated conditions (Fig. 6*D*) with the interaction being enhanced during serum stimulation, suggesting that prosurvival stimulation strengthens the association between TAp73 and c-Jun at the chromatin level. In contrast, their interaction at the chromatin level was significantly reduced upon UV treatment, implying that proapoptotic stress signals can lead to dissociation of TAp73 and c-Jun (Fig. 6*E*). Consistently, activation of the collagenase or cyclin D1 promoter-luciferase construct by TAp73 and c-Jun was reduced upon UV irradiation (Fig. 6*F*), although activation of the *mdm2* promoter-luciferase construct was not affected (data not shown), suggesting that disruption of the interaction between TAp73 and c-Jun during the stress response leads to compromised activation of AP-1 target genes.

Stresses like UV irradiation and stimulation with growth factors are known to induce phosphorylation of c-Jun (45, 46). Although JNK-dependent phosphorylation of c-Jun was not required for activation of the AP-1 target promoters as well as for cooperation with TAp73, TAp73 could induce phosphorylation of the c-Jun^D^ mutant (Fig. 1). We therefore wanted to assess whether c-Jun phosphorylation is required for binding to TAp73. To this end, we utilized the FLAG-tagged c-Jun^AA^ mutant that cannot be phosphorylated at serines 63 and 73. Isolation of chromatin-bound FLAG-tagged c-Jun^AA^ mutant or wild-type c-Jun and co-immunoprecipitation showed that the c-Jun^AA^ mutant was able to bind to TAp73 at the chromatin level (Fig. 6*G*), highlighting that phosphorylation of c-Jun was not necessary for binding to TAp73.

**FIGURE 7.**
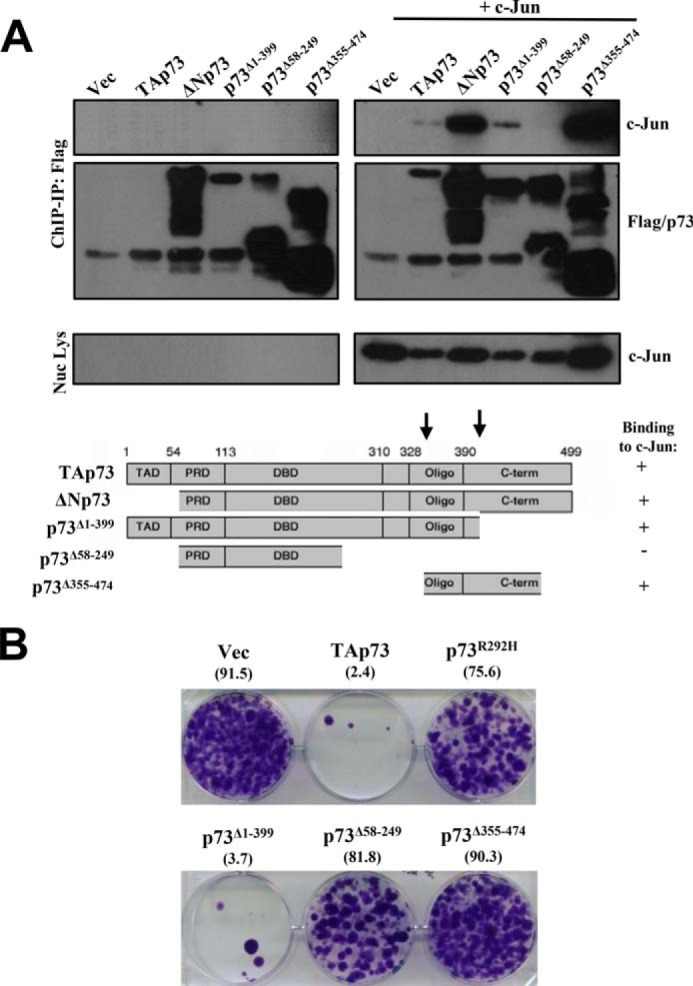
**C-terminal region of p73 is required for interaction with c-Jun.** *A*, similar chromatin immunoprecipitation (*IP*) experiments were carried out with various deletions mutants of FLAG-tagged TAp73β in H1299 cells as indicated in the absence or presence of c-Jun. A schematic summarizing the binding of the TAp73β mutants is shown below. *TAD*, transactivation domain; *DBD*, DNA-binding domain; *PRD*, proline-rich domain. *B*, H1299 cells were transfected with the different TAp73β deletion constructs, and cell survival was determined by growth of cellular colonies over 10–14 days in culture. Experiments were repeated at least two times, and representative results are shown. *Numbers* in *parentheses* indicates percent growth area. *Nuc Lys*, nuclear lysate; *Vec*, vector.

To delineate the region of p73 required for binding to c-Jun, we utilized several deletion mutants of p73 and assessed their binding to c-Jun at the chromatin level. Absence of the amino-terminal domain did not affect binding to c-Jun (Figs. 6, *B* and *C*, and 7*A*). Similarly, interaction with c-Jun was unaffected by the absence of the extreme carboxyl-terminal domain (*i.e.* TAp73^1–399^) (Fig. 7*A*). However, the deletion mutant partially lacking the DNA-binding domain and the rest of the carboxyl-terminal domain (*i.e.* TAp73^58–249^) was unable to bind c-Jun, whereas the carboxyl-terminal domain alone (*i.e.* TAp73^355–474^) could bind to c-Jun. These data thus indicate that the region between amino acids 355 and 399 of TAp73 is important for binding to c-Jun at the level of the chromatin. The deletion mutants of TAp73 were also tested for their effect on cellular survival by colony formation assay (Fig. 7*B*). Overexpression of TAp73 and TAp73^1–399^ led to a potent inhibition of cellular growth, whereas the DNA binding mutant TAp73^R292H^ did not. Similar to TAp73^R292H^, p73^58–249^ and p73^355–474^ did not induce growth arrest. However, p73^355–474^ expression marginally but consistently led to better survival than p73^58–249^ probably because of the ability of the former to interact with c-Jun.

##### Fos Family Members Are Recruited to the AP-1 Promoters in a c-Jun-dependent Manner upon TAp73 Expression and Cooperate with TAp73 to Potentiate Cellular Growth

**FIGURE 8.**
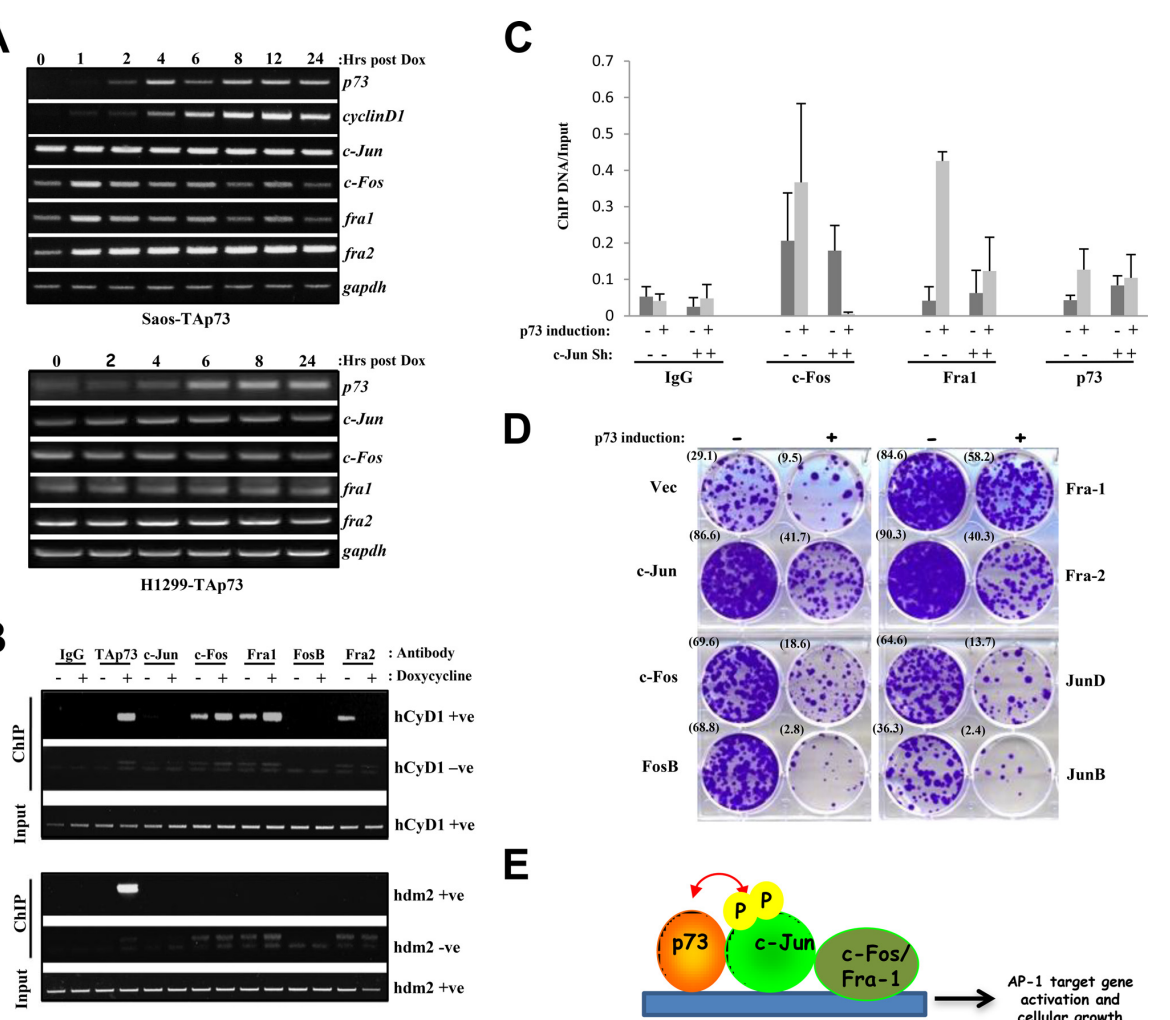
**TAp73 expression leads to the recruitment of AP-1 family members to target gene promoters in a c-Jun-dependent manner and potentiates cellular growth.** *A*, RNA was extracted from Saos2 or H1299-TAp73β inducible cells after induction with doxycycline (*Dox*). Semiquantitative PCR for target genes and AP-1 members was performed. *B*, Saos-TAp73β inducible cells were induced with doxycycline and subjected to chromatin immunoprecipitation with the control IgG or with antibodies against p73 or specific AP-1 members followed by semiquantitative PCR amplification of target regions on cyclin D1 or *mdm2* promoters. PCR primer set *hCyD1* +*ve* covers the region around the AP-1 site on the cyclin D1 promoter (as in Fig. 5*B*), whereas *hCyD1* −*ve* is the negative control. PCR primer set *hdm2* +*ve* is at the canonical binding site for TAp73 on the *mdm2* promoter, whereas *hdm2* −*ve* is the negative control. *C*, similar experiments were carried out in Saos-TAp73β inducible cells either in the absence or presence of c-Jun shRNA (*Sh*). Chromatin immunoprecipitation was carried out with the indicated antibodies followed by real time PCR analysis with the PCR primer set *hCyD1* +*ve* covering the AP-1 site on the cyclin D1 promoter. Data show the mean of experiments, and *error bars* indicate S.D. *D*, Saos-TAp73β inducible cells were stably transfected with the different AP-1 members, TAp73β expression was induced by doxycycline, and cell survival was determined by growth of cellular colonies over 10–14 days in culture. Representative pictures are shown from three independent experiments for all cases. *Numbers* in *parentheses* indicate percent growth area. *E*, working model. c-Jun is required for TAp73β-mediated AP-1 target gene activation in a manner dependent on the canonical AP-1 sites and the presence of Fos family members (c-Fos and Fra1) that dimerize with c-Jun. TAp73β does not transcriptionally activate AP-1 members but enhances c-Jun phosphorylation (33) and leads to the recruitment of Fos family members to the AP-1 target gene promoters. TAp73β binds to the promoters near the AP-1 site and interacts with c-Jun through its C-terminal region at the level of chromatin. This results in activation of AP-1 target genes and potentiation of cellular growth.

One of the major questions involving TAp73 and AP-1 members is how does TAp73 activate or potentiate the AP-1 response? To address this, we examined whether TAp73 can transcriptionally up-regulate the AP-1 members. Inducible expression of TAp73 in p53-null Saos2 or in H1299 cells induced AP-1 target genes such as cyclin D1 at the transcriptional level but did not regulate the levels of c-Jun, c-Fos, Fra1, and Fra2, suggesting that TAp73 does not regulate the AP-1 proteins (Fig. 8*A*). We therefore examined whether TAp73 induction would lead to the recruitment of AP-1 members to the AP-1 site on the target promoters. Chromatin immunoprecipitation assays done in Saos2-TAp73 inducible cells showed that upon induction of TAp73 there was an increase in c-Fos and Fra1 but not c-Jun, FosB, or Fra2 recruitment at the AP-1 sites on the endogenous cyclin D1 promoter (Fig. 8*B*). This was specific to the AP-1 target cyclin D1 promoter and not the canonical p53 target *mdm2* promoter on which only TAp73 was bound. The TAp73-mediated recruitment of c-Fos and Fra1 to the AP-1 site was dependent on c-Jun because depletion of c-Jun led to a marked decrease in the recruitment of c-Fos and Fra1 onto the cyclin D1 promoter (Fig. 8*C*), indicating that TAp73 indeed promotes the recruitment of AP-1 member in a c-Jun-dependent manner to the AP-1 target gene promoters.

Analysis of the functional relevance of the interaction between p73 and the AP-1 members by colony formation assay showed that although TAp73 induction alone led to decreased colony growth in Saos2 cells as expected there was a significant increase in cellular survival as determined by colony formation in the presence of c-Jun, c-Fos, and Fra1 (Fig. 8*D*, colony numbers indicated in *parentheses*). However, this was not the case in the presence of JunB, JunD, and FosB, which were also not recruited to AP-1 target promoters (Fig. 8*B* and data not shown) and which do not generally synergize as well as c-Fos or Fra1 with TAp73 to transactivate AP-1 target genes. Surprisingly, Fra2 also showed a cooperative effect with TAp73 on cellular growth, although it does not synergize with p73 in activating AP-1 target genes, nor is it required for p73-mediated target gene activation. These data therefore highlight the biological effects of cooperation between TAp73 and the selected AP-1 members on cellular growth.

## Discussion

The results presented here provide mechanistic insights into the role of TAp73 in transactivating AP-1 target genes and consequently promoting cellular growth. We provide evidence demonstrating that 1) TAp73 is able to cooperate with other selected AP-1 family members such as c-Fos and Fra1 besides c-Jun in transactivating AP-1 target genes and promoting cellular growth; 2) TAp73 is capable of binding to the AP-1 target promoters on or near the canonical AP-1 binding sites, a requirement for TAp73-mediated target gene activation; 3) TAp73 associates with c-Jun in the context of the chromatin; 4) its expression leads to recruitment of c-Fos and Fra1 onto the AP-1 target gene promoters; and 5) all these effects are dependent on the presence of c-Jun. Thus, TAp73 works in a complex with c-Jun and selected AP-1 family members to transactivate AP-1 target gene expression.

Although TAp73 has been demonstrated to be able to induce apoptosis and functions to suppress tumorigenesis, it is also overexpressed in many cancers. Our previous work had suggested a role for TAp73 in supporting cellular growth through the activation of AP-1 target genes such as cyclin D1 in a c-Jun-dependent manner (33). This role of TAp73 in promoting cellular growth has now been confirmed with the newly identified role for TAp73 in activating the pentose phosphate pathway and thus cellular proliferation (25). Hence, it is apparent that TAp73 can indeed promote cellular survival in a context-dependent manner especially in cancers where it is overexpressed. One context in which the growth-supporting role of TAp73 is manifested is when it was co-expressed with the AP-1 member c-Jun, leading to the potentiation of the expression of AP-1 targets such as cyclin D1, although further details had not been previously elucidated. Hence, we embarked on exploring the mechanistic details, and our studies have now revealed that other selected AP-1 members like c-Fos and Fra1 are also able to cooperate with TAp73 in a c-Jun-dependent manner.

AP-1 family proteins are transcription factors involved in a plethora of cellular process including apoptosis and growth promotion (28). Animal models have shown a tumor-promoting role for c-Jun, c-Fos, and Fra1. For instance, c-Jun and c-Fos cooperate to enhance c-Fos-mediated osteosarcoma formation (47), and c-Fos was shown to be required for development of 12-*O*-tetradecanoylphorbol-13-acetate-induced malignant skin tumors (48). Moreover, like c-Jun and c-Fos, Fra1 is also overexpressed in tumors (49), and in response to Ras expression, Fra1 was reported to cooperate with the Erk pathway and stabilize c-Jun by heterodimerizing with it (50). Thus, ample evidence exists for the promotion of tumor formation by these AP-1 factors, which are able to cooperate with TAp73 in promoting cellular growth. Incidentally, as overexpression of TAp73 has been noted in a large number of cancers (10, 11), it is tempting to speculate that the ability of TAp73 to promote cellular growth might be in these contexts due to co-expression with AP-1 proteins *in vivo*; this requires future investigation.

The cooperativity on target gene transactivation is consistent with the ability of TAp73 to bind to the chromatin near or at the AP-1 sites as assessed by chromatin immunoprecipitation assays. Although direct DNA binding was earlier ruled out by gel shift analysis using consensus AP-1 sites (33), the current data are consistent with TAp73 being bound to multiple sites on the genome at or close to AP-1 sites as also established by an earlier genome-wide chromatin immunoprecipitation study (51). Interestingly, the TAp73^R292H^ mutant that is incapable of binding to DNA is also defective in its ability to activate AP-1 target genes, further suggesting that the DNA binding ability of TAp73 is necessary for AP-1 target gene transactivation probably through c-Jun, which is required for the process. In this context, it is also to be noted that TAp73 was found to bind to c-Jun only in the context of the chromatin. Although it had been difficult to detect reciprocal interactions between p73 and c-Jun previously using cytosolic fractions (Fig. 6*A*), binding of TAp73 and c-Jun at or near the AP-1 sites suggests that TAp73 and c-Jun are in a complex at the level of chromatin.

Although the AP-1 sites are critical for TAp73-mediated transactivation of AP-1 target genes, other regulatory regions also appear to play a role. For example, the CRE sites on the cyclin D1 promoter appear to have a contributory role in TAp73-mediated activation. p300 and its homologue CRE-binding protein are known to bind to the CRE sites at the promoter of their target genes, interact with several transcription factors, and activate transcription (52). p300 has also been shown to bind to TAp73 and positively regulate its transcriptional activity (53). Thus, the possibility exists that p300 binds to the CRE site on the cyclin D1 promoter and enhances TAp73 activity at the AP-1 site. Conversely, TAp73 could influence the activity of the p300·CRE-binding protein complex, thereby activating the cyclin D1 promoter. Nonetheless, these cofactors appear to be specific for the individual target genes as CREs are absent on the collagenase promoter.

Similarly, although c-Jun is required for TAp73-mediated AP-1 target gene activation, it is also apparent that this occurs with other partners, especially c-Fos and Fra1, which are recruited to AP-1 target promoters in a c-Jun-dependent manner for the activation of targets, suggesting that TAp73 activation could lead to changes that result in this recruitment. Interestingly, although JunB alone did not cooperate with TAp73 in MEFs (Fig. 3*A*), it is noteworthy that JunB can substitute for c-Jun in mouse development and cell proliferation (54). Thus, JunB could possibly substitute for c-Jun in c-Jun^−/−^ MEFs, leading to some level of cooperation between c-Fos/Fra1 and TAp73 even in the absence of c-Jun as noted earlier (Fig. 3*A*). Surprisingly, however, although FosB and JunB did synergize to some extent with TAp73 in activating target genes, they were not recruited to the promoter upon TAp73 induction. This could possibly be due to context-specific effects as noted earlier. Thus, FosB and JunB might function differently compared with c-Fos and Fra1 perhaps by enhancing the activity of TAp73 on target gene promoters in contrast to c-Fos and Fra1, which are recruited to the promoters and thereby synergize with TAp73 in many different cell types.

In addition, we had previously found c-Jun to be phosphorylated in a JNK-independent manner by TAp73 upon its induction and that this phosphorylation was critical for the manifestation of the ability of TAp73 to transactivate AP-1 target genes (33). Consistently, the c-Jun^D^ mutant was phosphorylated in the presence of TAp73 and could cooperate with it (Fig. 1), but this phosphorylation is not required for binding at the chromatin level (Fig. 6*G*). Although the identity and mechanisms of regulators of TAp73-mediated c-Jun phosphorylation have yet to be unraveled, it is emerging that TAp73, which is overexpressed in cancers, causes c-Jun phosphorylation and thus could result in the recruitment of specific AP-1 members that would altogether cooperate in the activation of AP-1 target genes (Fig. 8*E*). Consistently, these AP-1 members were also found to be able to cooperate with TAp73 to promote cellular growth, highlighting the biological significance of these findings. These data are also conceptually similar to recent findings that AP-1 target genes are also similarly co-activated by other transcription factors such as FOXK2 in concert with AP-1 family members (55). In this context, it is noteworthy that treatment with DNA-damaging/genotoxic agents like UV irradiation led to a decrease in the interaction between c-Jun and TAp73 at the chromatin as well as activation of AP-1 target genes, and contrastingly, growth factor stimulation led to an increased association between them. This suggests that, during stress conditions, these two proteins may be less prone to interact and activate cell survival genes. Instead, TAp73 probably dissociates from c-Jun to induce apoptotic genes; this requires further investigation.

Taken together, the results presented demonstrate that TAp73 cooperates with several AP-1 members that dimerize to regulate AP-1 target gene transactivation and thus cellular growth. Thus, co-activation of TAp73 in the presence of AP-1 members would therefore provide a context for the manifestation of the survival properties of TAp73, which would otherwise lead to inhibition of cellular growth. Hence, these data suggest that inhibition of TAp73/AP-1 cooperation may be an avenue to inhibit tumor cell growth, especially in cancers that overexpress TAp73.

## Author Contributions

D. S. designed and performed most of the experimental work. W. B. designed and performed experiments shown in Fig. 8, *B–D*. K. S. conceived and designed the experiments and wrote the manuscript with input from D. S.

## Footnotes

2 The abbreviations used are:

AP: activator protein
MEF: mouse embryonic fibroblast
TRE: 12-*O*-tetradecanoylphorbol-13-acetate-responsive element
CRE: cyclic AMP-responsive element
ΔCol: minimal collagenase
IP: immunoprecipitation.

## References

[1] Levrero M. De Laurenzi V. Costanzo A. Gong J. Wang J. Y. Melino G. (2000) The p53/p63/p73 family of transcription factors: overlapping and distinct functions. J. Cell Sci. 113, 1661–1670 10769197 10.1242/jcs.113.10.1661

[2] Moll U. M. Slade N. (2004) p63 and p73: roles in development and tumor formation. Mol. Cancer Res. 2, 371–386 15280445

[3] Dötsch V. Bernassola F. Coutandin D. Candi E. Melino G. (2010) p63 and p73, the ancestors of p53. Cold Spring Harb. Perspect. Biol. 2, a004887 20484388 10.1101/cshperspect.a004887 PMC2926756

[4] Gong J. G. Costanzo A. Yang H. Q. Melino G. Kaelin W. G. Jr. Levrero M. Wang J. Y. (1999) The tyrosine kinase c-Abl regulates p73 in apoptotic response to cisplatin-induced DNA damage. Nature 399, 806–809 10391249 10.1038/21690

[5] Zaika A. Irwin M. Sansome C. Moll U. M. (2001) Oncogenes induce and activate endogenous p73 protein. J. Biol. Chem. 276, 11310–11316 11115495 10.1074/jbc.M005737200

[6] Lin K. W. Nam S. Y. Toh W. H. Dulloo I. Sabapathy K. (2004) Multiple stress signals induce p73β accumulation. Neoplasia 6, 546–557 15548364 10.1593/neo.04205 PMC1531659

[7] Jost C. A. Marin M. C. Kaelin W. G. Jr. (1997) p73 is a simian p53 related protein that can induce apoptosis. Nature 389, 191–194 9296498 10.1038/38298

[8] Zhu J. Jiang J. Zhou W. Chen X. (1998) The potential tumor suppressor p73 differentially regulates cellular p53 target genes. Cancer Res. 58, 5061–5065 9823311

[9] Hollstein M. Sidransky D. Vogelstein B. Harris C. C. (1991) p53 mutations in human cancers. Science 253, 49–53 1905840 10.1126/science.1905840

[10] Stiewe T. Pützer B. M. (2002) Role of p73 in malignancy: tumor suppressor or oncogene? Cell Death Differ. 9, 237–245 11859406 10.1038/sj.cdd.4400995

[11] Melino G. De Laurenzi V. Vousden K. H. (2002) p73: friend or foe in tumorigenesis. Nat. Rev. Cancer 2, 605–615 12154353 10.1038/nrc861

[12] Donehower L. A. Harvey M. Slagle B. L. McArthur M. J. Montgomery C. A. Jr. Butel J. S. Bradley A. (1992) Mice deficient for p53 are developmentally normal but susceptible to spontaneous tumors. Nature 356, 215–221 1552940 10.1038/356215a0

[13] Tomasini R. Tsuchihara K. Wilhelm M. Fujitani M. Rufini A. Cheung C. C. Khan F. Itie-Youten A. Wakeham A. Tsao M. S. Iovanna J. L. Squire J. Jurisica I. Kaplan D. Melino G. Jurisicova A. Mak T. W. (2008) TAp73 knockout shows genomic instability with infertility and tumor suppressor functions. Genes Dev. 22, 2677–2691 18805989 10.1101/gad.1695308 PMC2559903

[14] Yang A. Walker N. Bronson R. Kaghad M. Oosterwegel M. Bonnin J. Vagner C. Bonnet H. Dikkes P. Sharpe A. McKeon F. Caput D. (2000) p73-deficient mice have neurological, pheromonal and inflammatory defects but lack spontaneous tumours. Nature 404, 99–103 10716451 10.1038/35003607

[15] Lee C. W. La Thangue N. B. (1999) Promoter specificity and stability control of the p53-related protein p73. Oncogene 18, 4171–4181 10435630 10.1038/sj.onc.1202793

[16] Ishimoto O. Kawahara C. Enjo K. Obinata M. Nukiwa T. Ikawa S. (2002) Possible oncogenic potential of ΔNp73: a newly identified isoform of human p73. Cancer Res. 62, 636–641 11830511

[17] Zaika A. I. Slade N. Erster S. H. Sansome C. Joseph T. W. Pearl M. Chalas E. Moll U. M. (2002) ΔNp73, a dominant negative inhibitor of wild-type p53 and TAp73, is up-regulated in human tumors. J. Exp. Med. 196, 765–780 12235210 10.1084/jem.20020179 PMC2194062

[18] Wilhelm M. T. Rufini A. Wetzel M. K. Tsuchihara K. Inoue S. Tomasini R. Itie-Youten A. Wakeham A. Arsenian-Henriksson M. Melino G. Kaplan D. R. Miller F. D. Mak T. W. (2010) Isoform specific p73 knockout mice reveal a novel role for ΔNp73 in the DNA damage response pathway. Genes Dev. 24, 549–560 20194434 10.1101/gad.1873910 PMC2841333

[19] Novak U. Grob T. J. Baskaynak G. Peters U. R. Aebi S. Zwahlen D. Tschan M. P. Kreuzer K. A. Leibundgut E. O. Cajot J. F. Tobler A. Fey M. F. (2001) Overexpression of the p73 gene is a novel finding in the high-risk B-cell chronic lymphocytic leukemia. Ann. Oncol. 12, 981–986 11521806 10.1023/a:1011153206003

[20] Zwahlen D. Tschan M. P. Grob T. J. Peters U. R. Fink D. Haenggi W. Altermatt H. J. Cajot J. F. Tobler A. Fey M. F. Aebi S. (2000) Differential expression of p73 splice variants and protein in benign and malignant ovarian tumours. Int. J. Cancer 88, 66–70 10962441

[21] Conforti F. Yang A. L. Agostini M. Rufini A. Tucci P. Nicklison-Chirou M. V. Grespi F. Velletri T. Knight R. A. Melino G. Sayan B. S. (2012) Relative expression of TAp73 and ΔNp73 isoforms. Aging 4, 202–205 22388545 10.18632/aging.100441 PMC3348480

[22] Toh W. H. Logette E. Corcos L. Sabapathy K. (2008) TAp73β and DNp73β activate the expression of the pro-survival caspase 2S. Nucleic Acids Res. 36, 4498–4509 18611950 10.1093/nar/gkn414 PMC2490756

[23] Toh W. H. Kyo S. Sabapathy K. (2005) Relief of p53-mediated telomerase suppression by p73. J. Biol. Chem. 280, 17329–17338 15734740 10.1074/jbc.M500044200

[24] Lefkimmiatis K. Caratozzolo M. F. Merlo P. D'Erchia A. M. Navarro B. Levrero M. Sbisa' E. Tullo A. (2009) p73 and p63 sustain cellular growth by transcriptional activation of cell cycle progression genes. Cancer Res. 69, 8563–8571 19861536 10.1158/0008-5472.CAN-09-0259

[25] Du W. Jiang P. Mancuso A. Stonestrom A. Brewer M. D. Minn A. J. Mak T. W. Wu M. Yang X. (2013) TAp73 enhances the pentose phosphate pathway and supports cell proliferation. Nat. Cell Biol. 15, 991–1000 23811687 10.1038/ncb2789 PMC3733810

[26] Toh W. H. Siddique M. M. Boominathan L. Lin K. W. Sabapathy K. (2004) c-Jun regulates the stability and activity of the p53 homologue, p73. J. Biol. Chem. 279, 44713–44722 15302867 10.1074/jbc.M407672200

[27] Jochum W. Passegué E. Wagner E. F. (2001) AP-1 in mouse development and tumorigenesis. Oncogene 20, 2401–2412 11402336 10.1038/sj.onc.1204389

[28] Shaulian E. Karin M. (2002) AP-1 as a regulator of cell life and death. Nat. Cell Biol. 4, E131–E136 11988758 10.1038/ncb0502-e131

[29] Schreiber M. Kolbus A. Piu F. Szabowski A. Möhle-Steinlein U. Tian J. Karin M. Angel P. Wagner E. F. (1999) Control of cell cycle progression by c-Jun is p53 dependent. Genes Dev. 13, 607–619 10072388 10.1101/gad.13.5.607 PMC316508

[30] Shaulian E. Schreiber M. Piu F. Beeche M. Wagner E. F. Karin M. (2000) The mammalian UV response: c-Jun induction is required for exit from p53-imposed growth arrest. Cell 103, 897–907 11136975 10.1016/s0092-8674(00)00193-8

[31] Estus S. Zaks W. J. Freeman R. S. Gruda M. Bravo R. Johnson E. M. Jr. (1994) Altered gene expression in neurons during programmed cell death: identification of c-Jun as necessary for neuronal apoptosis. J. Cell Biol. 127, 1717–1727 7798322 10.1083/jcb.127.6.1717 PMC2120296

[32] Hasenfuss S. C. Bakiri L. Thomsen M. K. Williams E. G. Auwerx J. Wagner E. F. (2014) Regulation of steatohepatitis and PPARγ signaling by distinct AP-1 dimers. Cell Metab. 19, 84–95 24411941 10.1016/j.cmet.2013.11.018 PMC4023468

[33] Vikhanskaya F. Toh W. H. Dulloo I. Wu Q. Boominathan L. Ng H.-H. Vousden K. H. Sabapathy K. (2007) p73 supports cellular growth through c-Jun-dependent AP-1 transactivation. Nat. Cell Biol. 9, 698–705 17496887 10.1038/ncb1598

[34] Herber B. Truss M. Beato M. Müller R. (1994) Inducible regulatory elements in the human cyclin D1 promoter. Oncogene 9, 1295–1304 8134134

[35] Passegué E. Wagner E. F. (2000) JunB suppresses cell proliferation by transcriptional activation of p16(INK4a) expression. EMBO J. 19, 2969–2979 10856241 10.1093/emboj/19.12.2969 PMC203376

[36] Dulloo I. Sabapathy K. (2005) Transactivation-dependent and -independent regulation of p73 stability. J. Biol. Chem. 280, 28203–28214 15919663 10.1074/jbc.M501702200

[37] Bunjobpol W. Dulloo I. Igarashi K. Concin N. Matsuo K. Sabapathy K. (2014) Suppression of acetylpolyamine oxidase by selected AP-1 members regulates DNp73 abundance: mechanistic insights for overcoming DNp73-mediated resistance to chemotherapeutic drugs. Cell Death Differ. 21, 1240–1249 24722210 10.1038/cdd.2014.41 PMC4085530

[38] Brown P. H. Kim S. H. Wise S. C. Sabichi A. L. Birrer M. J. (1996) Dominant-negative mutants of cJun inhibit AP-1 activity through multiple mechanisms and with different potencies. Cell Growth Diff. 7, 1013–1021 8853897

[39] Sprowles A. Wisdom R. (2003) Oncogenic effect of Δ deletion in v-Jun does not result from uncoupling Jun from JNK signaling. Oncogene 22, 498–506 12555063 10.1038/sj.onc.1206165

[40] Bakiri L. Matsuo K. Wisniewska M. Wagner E. F. Yaniv M. (2002) Promoter specificity and biological activity of tethered AP-1 dimers. Mol. Cell. Biol. 22, 4952–4964 12052899 10.1128/MCB.22.13.4952-4964.2002 PMC133900

[41] Shiozawa T. Miyamoto T. Kashima H. Nakayama K. Nikaido T. Konishi I. (2004) Estrogen-induced proliferation of normal endometrial glandular cells is initiated by transcriptional activation of cyclinD1 via binding of c-Jun to an AP-1 sequence. Oncogene 23, 8603–8610 15467760 10.1038/sj.onc.1207849

[42] White L. A. Brinckerhoff C. E. (1995) Two activator protein-1 elements in the matrix metalloproteinase-1 promoter have different effects on transcription and bind Jun D, c-Fos and Fra2. Matrix Biol. 14, 715–725 8785586 10.1016/s0945-053x(05)80014-9

[43] Sun Y. Wenger L. Brinckerhoff C. E. Misra R. R. Cheung H. S. (2002) Basic calcium phosphate crystals induce matrix metalloproteinase-1 through the Ras/mitogen-activated protein kinase/c-fos/AP-1/metalloproteinase 1 pathway. Involvement of transcription factor binding sites AP-1 and PEA-3. J. Biol. Chem. 277, 1544–1552 11682465 10.1074/jbc.M100567200

[44] Chen X. Zheng Y. Zhu J. Jiang J. Wang J. (2001) p73 is transcriptionally regulated by DNA damage, p53 and p73. Oncogene 20, 769–774 11314010 10.1038/sj.onc.1204149

[45] Hibi M. Lin A. Smeal T. Minden A. Karin M. (1993) Identification of an oncoprotein- and UV-responsive protein kinase that binds and potentiates the c-Jun activation domain. Genes Dev. 7, 2135–2148 8224842 10.1101/gad.7.11.2135

[46] Karin M. (1995) The regulation of AP-1 activity by mitogen-activated protein kinases. J. Biol. Chem. 270, 16483–16486 7622446 10.1074/jbc.270.28.16483

[47] Grigoriadis A. E. Schellander K. Wang Z. Q. Wagner E. F. (1993) Osteoblasts are target cells for transformation in c-Fos transgenic mice. J. Cell Biol. 122, 685–701 8335693 10.1083/jcb.122.3.685 PMC2119671

[48] Saez E. Rutberg S. E. Mueller E. Oppenheim H. Smoluk J. Yuspa S. H. Spiegelman B. M. (1995) c-Fos is required for malignant progression of skin tumors. Cell 82, 721–732 7545543 10.1016/0092-8674(95)90469-7

[49] Verde P. Casalino L. Talotta F. Yaniv M. Weitzman J. B. (2007) Deciphering AP-1 function in tumorigenesis: fra-ternizing on target promoters. Cell Cycle 6, 2633–2639 17957143 10.4161/cc.6.21.4850

[50] Talotta F. Mega T. Bossis G. Casalino L. Basbous J. Jariel-Encontre I. Piechaczyk M. Verde P. (2010) Heterodimerization with Fra-1 cooperates with the ERK pathway to stabilize c-Jun in response to the RAS oncoprotein. Oncogene 29, 4732–4740 20543861 10.1038/onc.2010.211

[51] Koeppel M. van Heeringen S. J. Kramer D. Smeenk L. Janssen-Megens E. Hartmann M. Stunnenberg H. G. Lohrum M. (2011) Cross talk between c-Jun and TAp73a/b contributes to the apoptosis-survival balance. Nucleic Acids Res. 39, 6069–6085 21459846 10.1093/nar/gkr028 PMC3152320

[52] Shiama N. (1997) the p300/CBP family: integrating signals with transcription factors and chromatin. Trends Cell Biol. 7, 230–236 17708951 10.1016/S0962-8924(97)01048-9

[53] Zeng X. Li X. Miller A. Yuan Z. Yuan W. Kwok R. P. Goodman R. Lu H. (2000) The N-terminal domain of p73 interacts with the CH1 domain of p300/CREB binding protein and mediates transcriptional activation and apoptosis. Mol. Cell. Biol. 20, 1299–1310 10648616 10.1128/mcb.20.4.1299-1310.2000 PMC85269

[54] Passegué E. Jochum W. Behrens A. Ricci R. Wagner E. F. (2002) JunB can substitute for Jun in mouse development and cell proliferation. Nat. Genet. 30, 158–166 11818961 10.1038/ng790

[55] Ji Z. Donaldson I. J. Liu J. Hayes A. Zeef L. A. Sharrocks A. D. (2012) The forkhead transcription factor FOXK2 promotes AP-1 mediated transcriptional regulation. Mol. Cell. Biol. 32, 385–398 22083952 10.1128/MCB.05504-11 PMC3255788

